# Assessment of a novel ophthalmology tele-triage system during the COVID-19 pandemic

**DOI:** 10.1186/s12886-021-02112-0

**Published:** 2021-09-24

**Authors:** Angelica C. Scanzera, Arthur Y. Chang, Nita Valikodath, Emily Cole, Joelle A. Hallak, Thasarat Sutabutr Vajaranant, Sage J. Kim, R. V. Paul Chan

**Affiliations:** 1 Department of Ophthalmology and Visual Sciences, Illinois Eye and Ear Infirmary, University of Illinois at Chicago, 1855 W. Taylor Street, Chicago, IL 60612 USA; 2 Division of Health Policy & Administration, School of Public Health, University of Illinois at Chicago, 1603 W. Taylor Street, Chicago, IL 60612 USA

**Keywords:** COVID-19, Triage, Telehealth, Tele-ophthalmology, Tele-triage, Health services research

## Abstract

**Background:**

In response to the COVID-19 pandemic, a web-based tele-triage system was created to prioritize in-person clinic visits and ensure safety at the University of Illinois at Chicago Department of Ophthalmology and Visual Sciences during a statewide shelter-in-place order. The aim of this study is to evaluate the impact of the tele-triage system on urgent visit volume and explore the characteristics of acute visit requests at a tertiary referral eye center.

**Methods:**

This retrospective study analyzed acute visit requests between April 6, 2020 and June 6, 2020. Descriptive statistics, chi-square tests, ANOVA, and bivariate logistic regression were used to compare variables with a *p*-value of 0.05.

**Results:**

Three hundred fifty-eight surveys were completed. Mean age was 49.7 ± 18.8 years (range 2–91). The majority of requests were determined as urgent (63.0%) or emergent (0.8%). Forty-nine patients had recent eye trauma (13.7%), and the most common reported symptoms were new onset eye pain (25.7%) and photophobia (22.9%). Most patients were self-referred (63.7%), though provider referral was more common in patients with symptoms of new onset lid swelling (*p* < 0.01), diplopia (*p* < 0.01), flashing lights (*p* = 0.02), or droopy eyelid (*p* < 0.01). Patients presenting with symptom onset within 48 h tended to be younger (45.8 years) versus those with symptom duration of 48 h to 1 week (49.6 years), or more than 1 week (52.6 years; *p* < 0.01).

**Conclusion:**

This novel tele-triage system screened out one-third of acute visit requests as non-urgent, which limited in-person visits during the initial shelter-in-place period of the pandemic. Tele-triage systems should be implemented in eye care practices for future emergency preparedness.

## Background

The rapid spread of coronavirus (COVID-19) has affected every aspect of the healthcare system and has changed the way eye care practitioners provide care. Following recommendations from the Centers for Disease Control and Prevention (CDC) and the American Academy of Ophthalmology (AAO) [1, 2], the University of Illinois at Chicago (UIC) Department of Ophthalmology and Visual Sciences cancelled all elective procedures and clinic visits during a statewide shelter-in-place order; however, access to urgent eye care became a priority, requiring new approaches to providing this care safely. Given the absence of a widely available, low-cost rapid diagnostic test for the coronavirus at the time, there was an immediate need for a screening process to minimize the spread of disease while continuing to provide urgent eye care.

Telemedicine in other subspecialties has proven to play a critical role in improving access to acute care, to reduce non-urgent in-person visits, and to decrease healthcare costs [3, 4]. Owing to these benefits, telemedicine has been increasingly utilized as a modality for care delivery across multiple specialties in recent years, but especially during the present pandemic, in which any reduction in non-urgent in-person visits can be highly valuable and beneficial [5, 6]. Additional studies have shown telemedicine to be a feasible option specifically for eye care [7].

Accordingly, we built a tele-triage system to ensure patient, staff, and provider safety, as previously reported [8]. All individuals requesting an acute same-day in person visit at the Illinois Eye and Ear Infirmary (IEEI), an eye clinic within an academic medical center, were required to complete a HIPAA-compliant, web-based survey via RedCAP describing their history and symptoms. Survey questions included patient age, new or established patient status, referral source, ocular history and symptoms, immunocompromised status, COVID-19 status, and interest in a telehealth visit if eligible. Provider-determined level of visit urgency included urgent, emergent, routine, telehealth, or other. Urgent patients were scheduled for a same day clinic visit, emergent patients were directed to the Emergency Department (ED), and routine visits were scheduled 3 months out. ‘Other’ included patients who were recommended a non-urgent visit within 3 months. Level of urgency was determined by following the departmental protocol triage stratification table [9]. Patients at risk for having COVID-19 included those with symptoms of shortness of breath, cough, fever, anosmia, or contact with a person with known COVID-19.

The goals of this tele-triage system were to limit the number of patients entering IEEI, reduce exposures, and isolate patients who were immunocompromised or otherwise at-risk for COVID-19. In this study, we sought to evaluate the impact of a novel tele-triage system implemented early in the pandemic on urgent visit volume, and to describe characteristics of acute visit requests at an academic tertiary referral center.

## Methods

This was a retrospective cross-sectional study. Following the statewide shelter-in-place order, the tele-triage system was implemented on April 6, 2020. The system was removed from use on June 6, 2020, in response to a variety of factors, including greater availability of personal protective equipment, a statewide mandate for face-coverings in public indoor spaces, and other measures to protect patients and clinicians from the spread of coronavirus [10]. As such, surveys of all individuals requesting an acute in-person clinic visit between April 6, 2020 and June 6, 2020 were reviewed. Surveys were created for the purpose of patient care, and Institutional Review Board approval from UIC was later received in order to analyze this data. As all submitted surveys were evaluated for urgent visits, the only inclusion criteria for this study was submission of the patient survey. All patients who filled out the tele-triage survey – and thus, all patients who requested an urgent in-person eye evaluation between April 6, 2020 and June 6, 2020 – were included in the study. No patient could visit the IEEI for an in-person urgent appointment without having first filled out the survey and received subsequent confirmation to proceed to the clinic. For improved analysis, level of urgency was grouped: “urgent” included those who were recommended a same-day visit at IEEI or directed immediately to the ED; all others were considered “non-urgent.”

The primary outcome of the study was to determine the impact of the tele-triage system on volume of urgent visits in the eye clinic, as measured by the relative reduction in patient load. Secondary outcomes of the study included age, presenting ocular symptoms, referral status, determined urgency of visit, and other demographic and clinical factors of interest. Survey data were collected and managed using REDCap electronic data capture tools hosted at the University of Illinois at Chicago. REDCap (Research Electronic Data Capture) is a secure, web-based software platform designed to support data capture for research studies [11, 12]. Data was exported directly from REDCap into the analysis software, SAS Institute Inc. 2018 (SAS 9.4M6, Cary, NC, USA). Descriptive statistics as well as chi-square, ANOVA, and bivariate logistic regression were used to compare variables. Chi-square tests were used to compare referral source (self or provider) by type of symptom. After confirming that age was normally distributed, one-way ANOVA was performed to compare patient age against duration of symptoms. Bivariate logistic regression was used to compare both urgency of visit by duration of symptoms, and presence of each individual symptom by duration of symptoms. A *p*-value of ≤ 0.05 was considered statistically significant.

## Results

### Patient reported age, symptoms, and ocular history

A total of 358 surveys were submitted. Mean age was 49.7 ± 18.8 years (range 2–91). Of those, 49 patients had recent eye trauma (13.7%). The most common reason for acute visit request was new onset eye pain (25.7%, *n* = 92), followed by new symptoms of photophobia (22.9%, *n* = 82), vision loss (17.9%, *n* = 64), eye redness (16.8%, *n* = 60), lid edema (15.4%, *n* = 55), floaters (13.7%, *n* = 49), flashing lights (9.8%, *n* = 35), new or worsening diplopia (5.9%, *n* = 21), droopy eyelid (5.9%, *n* = 21), or curtain over vision (2.8%, *n* = 10). Median and mean (± standard deviation) number of symptoms per patient were 3.00 and 3.22 ± 1.74, respectively (range 1–11). Of the 350 patients who responded, 36.0% reported onset of symptoms within 24–48 h, 20.3% within 48 h to 1-week, and 43.7% greater than 1 week. The majority of patients were self-referred (63.7%). Table 1 further describes patient reported history. Patients were more commonly referred by a provider compared with self-referral if they had symptoms of new onset lid swelling (*p* = 0.03), diplopia (*p* < 0.01), flashing lights (*p* = 0.02), or droopy eyelid (*p* < 0.01). Chi-square analysis of patient symptoms by referral source can be found in Table 2.

**Table 1:** Patient reported history

**Characteristic**	**n (%)**
Patient status:	
Established IEEI patient	177 (49.4)
New to IEEI, Established in health system	66 (18.4)
New to UI Health system	115 (32.1)
Referral source	
Self	228 (63.7)
Eye doctor	53 (14.8)
Emergency room physician	26 (7.3)
Primary care physician	44 (12.3)
Other provider	7 (2.0)
Symptom duration	
Within 24 to 48 h	126 (36.0)
One week	71 (20.3)
More than 1 week	153 (43.7)
Previous surgeries^a^	86 (24.0)
Surgery within 3 months^a^	21 (5.9)
Surgery type	
Corneal Transplant	16
Retina	18
Glaucoma	12
Cataract	38
Other/unknown	26
Follow-up determined by eye care provider	
Urgent visit at IEEI	229 (64.0)
Immediate referral to UI Health ED	3 (0.8)
Routine visit (3–6 months)	50 (14.0)
Telehealth	6 (1.7)
Other	70 (19.6)

^a^In the same eye

**Table 2:** Chi-square analysis of patient symptoms by referral source

	Referral Source				*P*
	Self		Provider		
	N	(%)	N	(%)	
Trauma					
No	202	88.6	107	82.3	0.1
Yes	26	11.4	23	17.7	
Vision loss					
No	189	82.9	105	80.8	0.61
Yes	39	17.1	25	19.2	
Eye pain					
No	167	73.25	99	76.15	0.55
Yes	61	26.75	31	23.85	
Eye redness					
No	196	86	102	78.5	0.068
Yes	32	14	28	21.5	
Light sensitivity					
No	178	78.1	98	75.4	0.56
Yes	50	21.9	32	24.6	
Lid swelling					
No	200	87.7	103	79.2	0.03*
Yes	28	12.3	27	27.8	
Diplopia					
No	223	97.8	114	87.7	< 0.01*
Yes	5	2.2	16	12.3	
Floaters					
No	199	87.3	110	84.6	0.48
Yes	29	12.7	20	15.4	
Flashes					
No	212	93	111	85.4	0.02*
Yes	16	7	19	14.6	
Curtain over vision					
No	223	97.8	125	96.15	0.36
Yes	5	2.2	5	3.85	
Droopy eyelid					
No	222	97.4	115	88.5	< 0.01*
Yes	6	2.6	15	11.5	

*statistically significant

Patients presenting with a symptom onset greater than 1 week tended to be older than those with duration of 48 h to 1 week or 24 to 48 h (*p* < 0.01; Table 3).

**Table 3:** Mean age by duration of symptoms

Symptom Duration	n	Mean Age (years)	*P*
24 to 48 h	126	48.8 + -18.8	< 0.01*
48 h to 1 week	71	49.6 + -19.0	
> 1 week	153	52.6 + -17.8	

*statistically significant

Of all requests, 11 patients (3.1%) reported having had a positive COVID-19 test result, and an additional 33 patients (9.2%) were considered at risk of having COVID-19. In addition, 103 patients reported being immunocompromised, while an additional 16 reported a history of lung disease. One patient was pregnant and three reported unknown pregnancy status.

### Determined urgency of visit

**Fig. 1:**
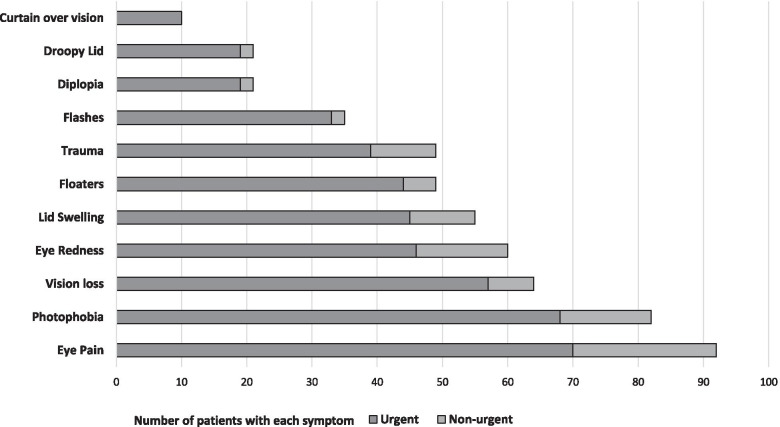
Presenting new symptoms and determined urgency of visit

**Fig. 2:**
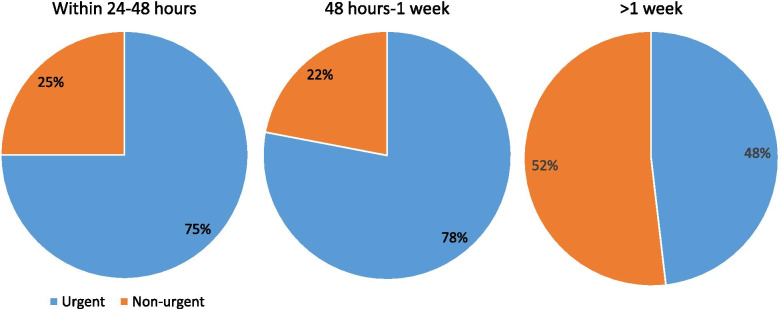
Onset of symptoms and determined urgency of visit

The majority of requests were determined as urgent (63.0%, *n* = 229) or emergent (0.8%, *n* = 3) by the evaluating eye care provider. No patient had repeat requests to attend a clinic visit having previously been scheduled to a non-urgent visit. Photophobia and eye pain were the most common symptoms and reasons for urgent visits. Figure 1 summarizes the frequency of reported symptoms by provider-determined level of urgency. When comparing each symptom by its duration, trauma was the only symptom that was statistically significant in which patients more often presented within 24–48 h of symptom onset (*p* = 0.01). Those with symptom duration less than 1 week were more often referred for urgent visit compared to those with symptoms greater than 1 week (*p* < 0.01; Fig. 2). About half of patients (48.3%) reported an interest in a telehealth visit, though only six patients were determined to be good candidates for a telehealth visit by the provider.

## Discussion

In this study, we assessed the use of a tele-triage system for acute visit requests during a statewide shelter-in-place order early in the COVID-19 pandemic. The key findings of this study are: 1) one-third of acute visit requests were able to be screened out as non-urgent using this tele-triage system, 2) patients were most commonly self-referred, though patients with symptoms of new onset eyelid swelling, diplopia, flashing lights, or droopy eyelid were more commonly referred by a provider, 3) patients presenting with a symptom onset greater than 1 week tended to be older, and 4) though almost half of the patients were open to telehealth visits, few were determined to be good candidates by the provider.

The first key finding of this study was that one-third of acute visit requests were screened out as non-urgent using this tele-triage system. While triage is often highlighted as a critical element in established guidelines, only a limited number of reports on specific methods and their effectiveness exist in the current literature. Bourdon et al. described the first 500 patients requesting emergency teleconsultation in Paris during the pandemic and reported a 73% reduction in patient volume by utilizing teleconsultation for all patients with ophthalmic concerns [13]. Similarly, by limiting our visits to urgent based on the previously discussed stratification, we saw an almost 90% reduction in patient volume in the first weeks of the pandemic, with the tele-triage system responsible for prioritizing acute visit requests and reducing in person urgent visits by one third. Kalra et al. used video visits to triage urgent cases and treat non-urgent pathology in an academic medical center [14]. Of 219 adult patients who had video encounters, 53% were routine visits and 47% were problem-focused visits. Though not stratified by problem-focused visits, 16% were triaged as high risk, 69% moderate, and 15% low risk. These studies demonstrate the successful use of triage systems in ophthalmology via virtual platforms in reducing cases that do not warrant in-person visits to the eye clinic or the ED.

Tele-triage models in other specialties have also demonstrated the effectiveness to screen out non-urgent visits prior to the pandemic. For example, Langabeer et al. initiated an emergency medical service telehealth model to avert non-urgent 911 calls to primary care clinics and reported a 6.7% reduction in unnecessary ED visits [15]. Frid et al. introduced a telemedicine program in Buenos Aires for patients with upper respiratory tract infections (URTI) during a flu outbreak in 2018 and prevented 98% of URTI related ED visits [16]. These promising findings show the potential of technology in triage systems to improve acute access to care for the patients who are most at need.

Although there remains no universal protocol for providing eye care during the pandemic, several ophthalmology departments implemented new protocols for screening acute visit requests. Risk assessment measures were adopted to stratify patients into three categories: 1) high risk requiring an immediate in-person visit, 2) medium risk requiring a telehealth or phone visit, or 3) low risk which can be rebooked at a later specified time [9, 17–19]. Use of this stratification in a triage system is crucial for consistency.

The second key finding was that patients in this study were more commonly referred by a provider, compared to self-referral, if they had symptoms of new onset lid swelling, diplopia, flashing lights, or droopy eyelid. One potential explanation is that primary care providers can correctly recognize the above symptoms as “red flags” for potential ophthalmic emergencies but may not be comfortable providing a diagnosis. Another hypothesis is that internet resources have provided the ability for individuals to triage their own health issues prior to seeing a healthcare provider, leading to a self-referral directly to the specialist. Studies have shown that 86.6% of patients use the internet for health purposes, and half will seek health care based on information from the internet [20]. Though online resources can be informative, they may not appropriately emphasize the urgency of these particular symptoms, and thus may not always make the best recommendation for seeking care.

The third key finding showed that the majority of patients in our study reported symptoms greater than 1 week prior to seeking care, and that the duration of symptoms increased with increasing age. Delay in medical care due to the pandemic has been reported across multiple medical subspecialties [21–24]. Our health system serves residents of communities on the south and west sides of Chicago, where almost half of the residents report a household income of less than $40,000 [25]. These communities were most directly affected by COVID-19 early in the pandemic, with coronavirus-related deaths clustered in predominantly African American communities on the south and west sides of Chicago [26]. Studies conducted prior to the pandemic found that financial distress was correlated with a delay in care regardless of health insurance status [27]. National rates of delaying necessary healthcare was near 25% in 2010 [28]. In our study, 43% of patients reported symptom onset over 1 week, suggesting that, in addition to other variables, factors related to the pandemic, such as financial distress and possibly fear due to proximity to individuals who died from COVID-19, are likely responsible for this delay. As this study only included data from the surveys which were created for the purpose of patient care, demographics were limited to age and new versus established patient status. Future studies should look further into the indirect effects of COVID-19 on health disparities.

A survey conducted by the Kaiser Family Foundation found that 48% of Americans reported that they or a family member “skipped or delayed medical care” because of the pandemic [29]. Similarly, in the study by Kalra et al., 49% of participants noted that they would have delayed seeking care if not for the availability of telehealth [30]. Our data only shows the patients who delayed care in the short term, but eventually sought out urgent care. The proportion of patients who delayed care beyond the short term is likely much greater than the 43% found in our study. We must consider the limitations of seeking care, such as fear or financial hardship, and drive policy to provide necessary care to our communities.

The fourth key finding showed that, although almost half of the patients in the present study reported interest in a telehealth visit, only a small number of patients were deemed appropriate for this visit type. In our system, telehealth encounters were suggested for medium risk cases; however, as telehealth video visits were only introduced in our department during the pandemic, providers likely had not yet adapted to the technology. In addition, a high proportion of patients were determined to warrant in-person evaluation, limiting telehealth as an option. The aforementioned Paris study strongly supports the use of telehealth for triage and treatment, given that a teleconsultation was sufficient for 73% of patients, and only 1% of patients experienced delayed care due to a misdiagnosis during the telehealth visit [13]. Furthermore, two other studies also reported favorable feedback, with 78% of participants in a study by Kalra et al. indicating they would consider opting for telehealth instead of a clinic visit in the future, and Gupta et al. reported that more than 88% of patients from a rheumatology clinic in India preferred teleconsultations over physical visits during the pandemic [14, 31]. These studies suggest that the use of video visits to triage patients in place of the current web-based system might provide more information and reduce the need for urgent in person visits.

Physician attitudes toward telehealth have also changed. Prior to the pandemic, although eye care providers had begun to acknowledge telemedicine as an important application in eye care, many had felt that current modalities still needed validation [32]. As the pandemic permanently changed the healthcare system, adapting to telemedicine became a necessity. A survey by the All India Ophthalmological Society during the pandemic revealed that 98.6% of clinicians had interest in adding tele-ophthalmology into their practice [33]. Another survey of neuro-ophthalmologists found an increase in telehealth utilization, with clinicians citing access, continuity, and patient efficiency of care as key benefits [34]. As such, telehealth was reported to be most useful for diagnoses based on history, external examination, and existing testing results [34]. Oculoplastic telemedicine consultations have also been recommended as a utility for follow-up and post-operative patients [35]. These studies suggest that telehealth may be more applicable in ophthalmic subspecialties which rely on imaging and external examination. Increased utilization of telehealth in the U.S. could also be attributed to changes in insurance reimbursement. The Centers for Medicare and Medicaid Services (CMS) expansion of video visits allowed patients to access their doctors without leaving home [36]. This is of particular importance, as the current CMS expansion and reimbursement model is scheduled to last through the public health emergency. In order for providers to commit to utilizing and investing in telehealth, measures are needed to permanently guarantee current telehealth reimbursement models and educate providers to maximize the quality of telehealth encounters. A study by De Lott et al. further demonstrates that clinician confidence in telemedicine is a key factor for sustained telemedicine utilization [37]. Of note, other challenges to the adoption of telemedicine in general, such as concerns over patient confidentiality, health disparities, and quality of care, certainly need to be addressed as well [38].

In addition to its primary purpose of triaging patients based on urgency, our system also allowed for safely identifying and isolating patients in need of urgent care immediately upon arrival to our clinic. Of all requests in our population, 11 patients were COVID-19 positive and an additional 33 patients were considered at risk of having COVID-19. In addition, 103 patients self-reported being immunocompromised, while an additional 16 reported history of lung disease. Identifying patients who may be immunocompromised or COVID-19 positive provided the opportunity to sequester these patients from others upon entry. This helped decrease the risk of provider and patient exposures in clinic.

Based on our experience and findings, we have discovered several limitations of this tele-triage system. We used a HIPAA compliant web-based system that did not integrate with electronic health records. This system also required a team approach, with two individuals committed to the system full-time, and up to 8 involved throughout each day. In addition, physicians involved in making the triage decision found that they called each patient and reviewed symptoms to better determine the urgency of the visit. With this knowledge, we would likely consider scheduling all acute visit requests as telehealth visits or moving to a data management system integrated with the patient’s medical record in the future. Additionally, with the advent of artificial intelligence (AI) applications, we will be able to develop automated systems that integrate triage data with patient metadata and classify patients into urgent and non-urgent, as suggested in previous studies [39].

There are a number of limitations in this study. 1) This study describes patient reported symptoms and history, and there is potential for recall bias; however, surveys were completed for the purpose of patient care while patients were experiencing active symptoms, so the risk of recall bias is likely to be low. Patients were also asked to select all symptoms that they were experiencing. In hindsight, selection of a primary symptom or ranking of all symptoms could have helped in better assessing urgency and understanding the association between symptoms. In practice, providers determined urgency based on survey data, discussion with the patient, and chart review. The latter could have influenced provider decision making, and we are unable to quantify the discrepancy in patient reported symptoms on the survey compared to symptoms elicited on phone discussion with the physician. 2) Though all providers were provided with the departmental risk assessment stratification table, variability in decision-making is likely based on clinical experience. Some authors suggest this variability might be due to the mentorship model in educating future clinicians [40–44]. Further studies are necessary to understand this variability in decision making in ophthalmology. 3) This study focused solely on survey data, which was created for the purpose of patient care, so any potential confounding effects of additional demographic or medical factors were unable to be determined. For the same reason, the outcomes of those who were determined to be non-urgent were not assessed. We did not have any record of a patient who presented to our institution’s emergency department after being triaged as non-urgent; however, we cannot confirm whether a patient went to an outside emergency department which represents a limitation of this system and other tele-triage systems. Future outcome studies are needed to better understand clinical outcomes. Moreover, when assessing delay in care, we only have data on the patients who did reach out for acute care, and we are unable to assess why patients who did not seek care may have delayed necessary care.

## Conclusion

In conclusion, our results show the utility of a web-based tele-triage system in screening out one-third of acute visit requests and limiting in-person visits to those that were urgent early in the COVID-19 pandemic. Patients were mostly self-referred, and provider referral was most commonly seen with patient-reported symptoms of new-onset lid swelling, diplopia, flashing lights, or droopy eyelid. Duration of symptoms at presentation increased with increasing age, with most patients presenting with symptoms greater than 1 week. Future studies are needed to evaluate the outcomes of urgent visits and understand the reason for delay in seeking care during the pandemic. We believe a technology driven triage system should be implemented for emergency preparedness.

## Data Availability

De-identified data are available from the corresponding author on reasonable request.

## Abbreviations

CDC: Centers for Disease Control and Prevention
AAO: American Academy of Ophthalmology
UIC: University of Illinois at Chicago
IEEI: Illinois Eye and Ear Infirmary
ED: Emergency Department
CMS: Centers for Medicare and Medicaid Services
AI: Artificial intelligence
